# Automated Cervical Nuclei Segmentation in Pap Smear Images Using Enhanced Morphological Thresholding Techniques

**DOI:** 10.3390/diagnostics15182328

**Published:** 2025-09-14

**Authors:** Wan Azani Mustafa, Khalis Khiruddin, Syahrul Affandi Saidi, Khairur Rijal Jamaludin, Halimaton Hakimi, Mohd Aminudin Jamlos

**Affiliations:** 1 Faculty of Electrical Engineering & Technology, Universiti Malaysia Perlis, Pauh Putra Campus, Arau 02600, Perlis, Malaysia; khalisdanial@studentmail.unimap.edu.my (K.K.); syahrulaffandi@unimap.edu.my (S.A.S.); 2 Advanced Computing (AdvComp), Centre of Excellence (CoE), Universiti Malaysia Perlis, Campus Pauh Putra, Arau 02600, Perlis, Malaysia; 3 Faculty of Artificial Intelligence, Universiti Teknologi Malaysia, Jalan Sultan Yahya Petra, Kuala Lumpur 54100, Malaysia; khairur.kl@utm.my; 4 Department of Computing, Institute of Emerging Digital Technologies, Universiti Teknologi PETRONAS, Persiaran UTP, Seri Iskandar 32610, Perak, Malaysia; 5 Faculty of Electronic Engineering & Technology, Universiti Malaysia Perlis, Pauh Putra Campus, Arau 02600, Perlis, Malaysia; mohdaminudin@unimap.edu.my

**Keywords:** nucleus segmentation, image quality assessment, adaptive morphological

## Abstract

**Background and Objective:** Cervical cancer remains one of the leading causes of death among women worldwide, particularly in regions with limited access to early screening. Pap smear screening is the primary tool for early detection, but manual interpretation is labor-intensive, subjective, and prone to inconsistency and misdiagnosis. Accurate segmentation of cervical cell nuclei is essential for automated analysis but is often hampered by overlapping cells, poor contrast, and staining variability. This research aims to develop an improved algorithm for accurate cervical nucleus segmentation to support automated Pap smear analysis. **Method:** The proposed method involves a combination of adaptive gamma correction for contrast enhancement, followed by Otsu thresholding for segmentation. Post-processing is performed using adaptive morphological operations to refine the results. The system is evaluated using standard image quality assessment metrics and validated against ground truth annotations. **Result:** The results show a significant improvement in segmentation performance over conventional methods. The proposed algorithm achieved a Precision of 0.9965, an F-measure of 97.29%, and an Accuracy of 98.39%. The PSNR value of 16.62 indicates enhanced image clarity after preprocessing. The method also improved sensitivity, leading to better identification of nuclei boundaries. Advanced preprocessing techniques, including edge-preserving filters and multi-Otsu thresholding, contributed to more accurate cell separation. The segmentation method proved effective across varying cell overlaps and staining conditions. Comparative evaluations with traditional clustering methods confirmed its superior performance. **Conclusions:** The proposed algorithm delivers robust and accurate segmentation of cervical cell nuclei, addressing common challenges in Pap smear image analysis. It provides a consistent framework for automated screening tools. This work enhances diagnostic reliability in cervical cancer screening and offers a foundation for broader applications in medical image analysis.

## 1. Introduction

Cervical cancer starts in the cells of the cervix, which is the lower part of the uterus connected to the vagina. It occurs when these cells grow abnormally, as highlighted by Guimarães et al. and Sausen et al. [1,2]. In 2020, there were approximately 604,000 new cases of cervical cancer and 342,000 deaths worldwide, according to Sausen et al. and Sung et al. [2,3]. Notably, this type of cancer is one of the most common among women and has a high death rate, as reported by Shi et al. [4]. A common test used to detect it is the Papanicolaou test, also known as the Pap smear, which was introduced in the 1940s. This test examines cervical cells under a microscope to identify early signs of cancer [5]. Although it has been widely employed, researchers such as [6,7] have highlighted that the Pap smear is imperfect. Due to its low sensitivity and specificity, it can occasionally miss cancer (false negatives) or give a false alarm (false positives).

Segmenting cells has become a crucial step in understanding how cervical cancer develops [8] mentioned that this need has led to more use of computer-aided detection tools to help with screening. Note that traditional manual methods are still applicable in places with limited medical resources. Even with improved technology, it is still challenging to obtain a reliable diagnosis, as noted by [9]. A key part of automated screening is segmenting the nuclei in Pap smear images. However, this is not easy [10] stated that problems like overlapping cells, false edges, and other image noise complicate this process. Due to these issues, it is still hard for automatic systems to correctly identify abnormal cells. Recently, artificial intelligence has become increasingly prevalent in this area since deep learning techniques can automatically select notable features in images with high accuracy and low error rates [11]. These methods are being used more and more in studies on cell segmentation. Correspondingly, this study aims to contribute to this area by proposing a better way to identify nuclei in cervical cell images.

In response to these challenges, recent studies have proposed various enhancement and segmentation techniques to improve performance. For example, adaptive gamma correction, Otsu thresholding, and mathematical morphological operations have improved contrast and boundary detection [12,13]. Bataineh [14] applied adaptive gamma correction for image brightness enhancement, while Nahrawi et al. [12] utilized morphological segmentation to achieve an accuracy of 93.66%. In addition, clustering algorithms such as Fuzzy C-means, K-means, and polynomial contrast enhancement have been explored for improved nucleus localization.

Recent deep learning-based approaches have further pushed the boundaries of segmentation accuracy. Tan et al. [15] proposed a deep ensemble model combining U-Net, U-Net++, DeepLabV3+, TransUNet, and SegFormer, achieving Dice coefficients up to 0.95 on cytoplasm segmentation. Meanwhile, Mustafa et al. [16] introduced a fuzzy rank-based late fusion strategy, integrating outputs from UNet, SegNet, and PSPNet to enhance segmentation performance on Herlev and JUCYT-v1 datasets. For cases with complex overlaps, Ding et al. [17] designed IMBMDCR-Net, a multi-branch deformable convolutional network that improved accuracy in overlapping cell detection. At the same time, Mahyari et al. [18] applied a multi-layer random walker with probabilistic deep learning to address boundary ambiguity and overlapping regions. Moreover, Liu et al. [19] proposed a Local Label Point Correction (LLPC) method to refine weak or inconsistent manual labels for better edge prediction.

Additional recent methods have also contributed significantly to cervical cell segmentation. For example, Li et al. [20] used a Deep Convolutional Neural Network (DCNN) to automatically segment cervical cells in Pap smear images, incorporating multi-scale feature fusion to oversee cells of different sizes. Their method achieved improved accuracy, particularly for images with overlapping cells. In addition, Ramakrishnan et al. [21] proposed a Hybrid CNN-RNN architecture for cervical cell segmentation, combining Convolutional Neural Networks (CNNs) for feature extraction with Recurrent Neural Networks (RNNs) for handling the sequential nature of cell structures. This method demonstrated potential in enhancing the model’s ability to identify irregularities in cell shapes and structures, improving segmentation results in challenging cases.

In addition, Zhao et al. [22] introduced a Graph-based Deep Learning Approach to improve the delineation of cell boundaries. By modeling the nuclei as graph nodes and using graph neural networks to learn the spatial relationships, their method provided more accurate and robust segmentation, particularly in datasets with dense or overlapping cells. Meanwhile, Ren et al. [23] implemented a Self-Supervised Learning (SSL) framework to reduce reliance on large annotated datasets. Their framework used a semi-supervised model, which trained the algorithm using labeled and unlabeled data, improving segmentation performance with limited annotated samples.

Unlike conventional thresholding methods that rely on fixed parameters and fail under uneven illumination or overlapping cells, the proposed approach introduces a hybrid framework that combines adaptive gamma correction, multi-Otsu thresholding, and adaptive morphological refinement. This design allows dynamic contrast enhancement and segmentation tailored to image-specific characteristics, improving boundary detection and reducing false positives. Compared to deep learning methods, our approach eliminates the dependency on large annotated datasets and high computational resources while still achieving competitive accuracy. These features make the proposed method a practical and innovative solution for automated cervical nucleus segmentation in resource-limited settings.

The remainder of this paper is organized as follows: Section 2 describes the proposed methodology in detail. Section 3 presents the experimental results and provides an in-depth discussion in Section 4. Finally, Section 5 concludes the study and contribution.

## 2. Materials and Methods

### 2.1. Algorithm Workflow

**Figure 1:**
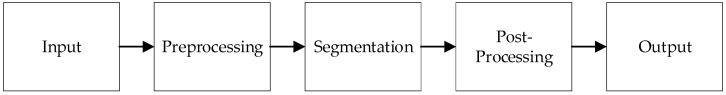
Block diagram implementation of cervical nucleus detection in this study.

Figure 1 displays the overall process for the developed enhanced segmentation algorithm. The algorithm uses a cervical Pap smear image from the Herlev dataset as input. For this project, 70 images were selected. The input image then undergoes preprocessing to enhance its quality and prepare it for accurate segmentation. Consequently, the processed image is segmented to isolate the areas of interest, specifically the cervical nuclei. Post-processing, which involves adaptive morphological operations, is applied to refine the segmented image. The final output is the segmented image, where cervical nuclei are clearly distinguished from the background.

### 2.2. Data Acquisition

**Figure 2:**
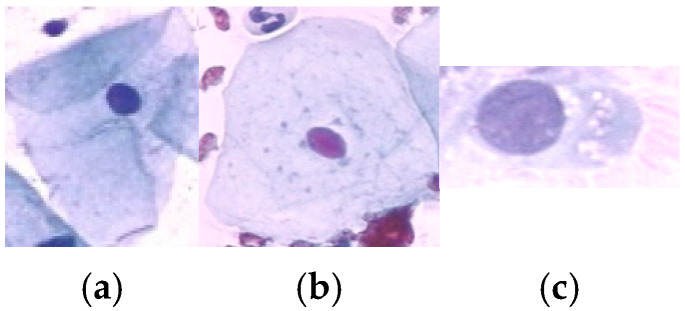
Sample data of Normal class: (**a**) Superficial Squamous Epithelia, (**b**) Intermediate Squamous Epithelia and (**c**) Columnar Epithelia.

**Figure 3:**
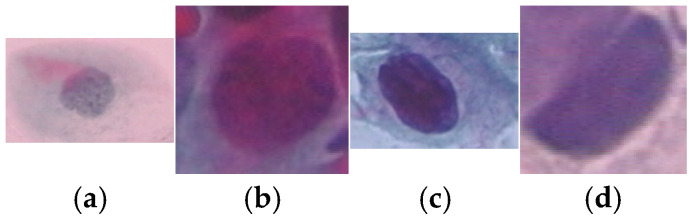
Sample data of abnormal class: (**a**) Mild Squamous Dysplasia, (**b**) Moderate Dysplasia, (**c**) Severe Dysplasia and (**d**) Carcinoma in Situ.

A total of 917 cervical cell images were obtained from the publicly available Herlev database, developed by Herlev University Hospital, Denmark, through the NiSIS EU coordination action (contract 13569). Note that this dataset is widely recognized and frequently used in numerous studies for evaluating cervical cell segmentation algorithms [24,25,26,27,28,29] due to its well-annotated and diverse set of cytological images. The dataset comprises images categorized into seven distinct classes, including three types of normal cells (242 images) and four types of abnormal cells (675 images). The normal cell classes include superficial squamous (74 images), intermediate squamous (70 images), and columnar epithelial cells (98 images). In addition, the abnormal cell categories include mild dysplasia (193 images), moderate dysplasia (146 images), severe dysplasia (197 images), and carcinoma in situ (150 images). Representative samples from each class are illustrated in Figure 2 and Figure 3.

From the Herlev dataset’s seven classes (3 normal: superficial squamous, intermediate squamous, columnar; 4 abnormal: mild, moderate, severe dysplasia, carcinoma in situ), we selected 70 images comprising N_normal = 30 and N_abnormal = 40 images (total 70). By subclass: superficial squamous = 10, intermediate squamous = 10, columnar = 10; mild dysplasia = 10, moderate dysplasia = 10, severe dysplasia = 10, carcinoma in situ = 10. Selection used stratified random sampling to mirror the source dataset’s imbalance while ensuring a minimum of m images per subclass. We report metrics both overall and by (normal vs. abnormal) groups to assess imbalance effects.

### 2.3. Image Preprocessing

**Figure 4:**
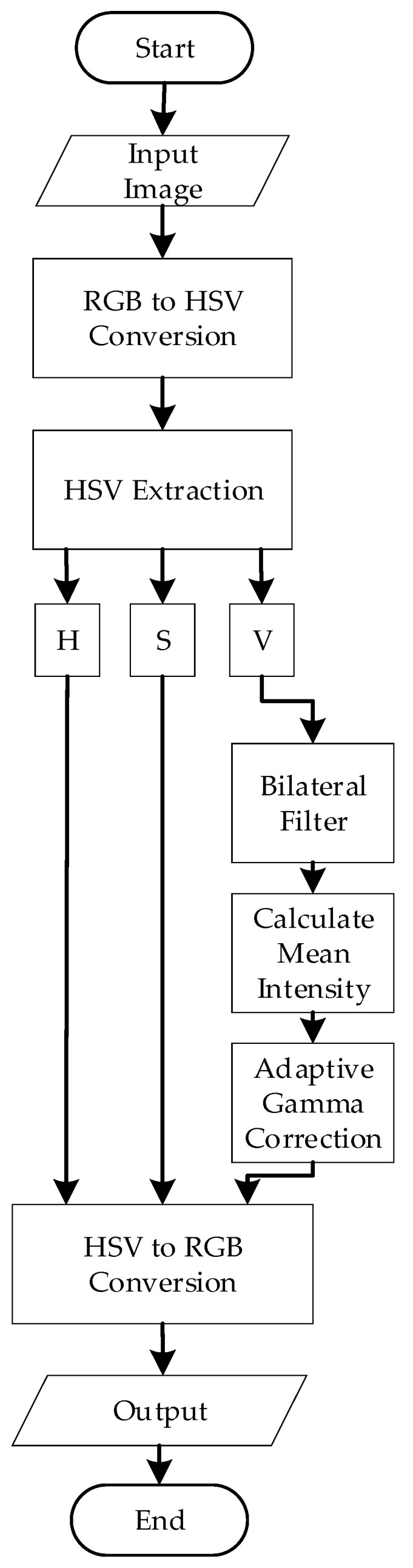
Preprocessing Flowchart.

Figure 4 illustrates the algorithm for preprocessing. The process begins by converting the input image $I_{RGB}$ from Red-Green-Blue (RGB) color space to the Hue-Saturation-Value (HSV) color space. This conversion is essential for enhancing contrast and separating chromatic components. The transformation from RGB to HSV can be mathematically represented by nonlinear functions that compute the hue *H*, saturation S, and value *V* channels based on the RGB intensities R,G,B ∈[0, 1]. Specifically, the Value channel *V* is computed as in Equation (1):

(1) V=max(R,G,B),

After conversion, the *V* channel is isolated, and bilateral filtering is applied. The bilateral filter is a nonlinear, edge-preserving, and noise-reducing smoothing filter that preserves sharp edges while effectively reducing noise. The bilateral filter combines two components: the Spatial Gaussian Filter and the Intensity Gaussian filter [30]. In particular, the Spatial Gaussian Filter ensures that the influence of nearby pixels decreases with distance from the target pixel.

On the other hand, the Intensity Gaussian filter ensures that pixels with similar intensities to the target pixel contribute more significantly to the result. This preserves the edges by reducing the impact of pixels with vastly different intensities. The mathematical representation of the bilateral filter for a pixel at location (x, y) in an image is represented in Equation (2):

(2) $$I^{\prime }\left(x,y\right)=\frac{1}{W(x,y)}\sum_{\left(i,j\right)\;\in \;S}I\left(i,j\right)\cdot G_{s}\left(\left\|\left(i,j\right)-\left(x,y\right)\right\|\right)\cdot G_{r}\left(\left\|I\left(i,j\right)-I\left(x,y\right)\right\|\right),$$

where $I\left(x,y\right)$ is the original intensity of the pixel, $I^{\prime }\left(x,y\right)$ is the filtered intensity, S is the spatial neighborhood, $G_{s}\left(\left\|\left(i,j\right)-\left(x,y\right)\right\|\right)$ is the spatial Gaussian component, $G_{r}\left(\left\|I\left(i,j\right)-I\left(x,y\right)\right\|\right)$ is the range (intensity) Gaussian component, and W is a normalization factor.

After bilateral filtering, the filtered V-channel now represents the intensity information about the image. To analyze the overall brightness of the image, the mean intensity was calculated using the formula in Equation (3):

(3) $$Mean\;Intensity=\;\;\frac{1}{M\times N}\sum_{i=1}^{M}\sum_{j=1}^{N}V\left(i,j\right),$$

where M×N are the dimensions of the image and $V\left(i,j\right)$ is the intensity value at the pixel $\left(i,j\right)$ of the filtered V-channel. Notably, darker images have lower mean intensity values, while brighter images exhibit higher values. In this project, images with an intensity greater than 0.5 are considered darker images, while images with an intensity equal to or less than 0.5 are considered overly bright. This measurement was critical for determining the gamma value adaptively.

Next, adaptive gamma correction is applied. In this project, the gamma value (γ) was defined adaptively based on the mean intensity of the filtered V-channel. The adaptive gamma adjustment was designed to brighten darker images using a gamma value greater than 1 and reduce the brightness of overly bright images using a gamma value less than 1. The mathematical equations are represented by Equations (4) and (5). For darker images (Mean intensity > 0.5):

(4) γ=1+(0.5 −Mean Intensity)

For brighter images (Mean intensity ≤ 0.5):

(5) $$\gamma =\frac{1}{2\;\times \;Mean\;Intensity}.$$

After applying adaptive gamma correction to the Value (*V*) channel in the HSV color space, the image is reconverted to RGB color space for further segmentation. This inverse conversion is based on the following intermediate calculations with the functional components of chroma (C), intensity adjustment (X), and brightness matching value (m) as shown in Equation (6)–(9):

(6) C=V·S

(7) H∈[0°, 360°]

(8) $$X=C\cdot \left(1-\left|\left(\frac{H}{60{}^{\circ}}\;mod\;2\right)-1\right|\right)$$

(9) m=V−C

Based on the sector in which H falls, the intermediate RGB values ($R^{\prime },G^{\prime },B^{\prime })$ are assigned as follows:

If $0{}^{\circ}\leq H<60{}^{\circ}:\left(R^{\prime },G^{\prime },B^{\prime }\right)=(C,X,0)$
If $60{}^{\circ}\;\leq H\;<120{}^{\circ}:\;\left(R^{\prime },G^{\prime },B^{\prime }\right)=(X,C,0)$
If $120{}^{\circ}\;\leq H\;<180{}^{\circ}:\;\left(R^{\prime },G^{\prime },B^{\prime }\right)=\left(0,C,X\right)$
If $180{}^{\circ}\;\leq H\;<240{}^{\circ}:\;\left(R^{\prime },G^{\prime },B^{\prime }\right)=\left(0,X,C\right)$
If $240{}^{\circ}\;\leq H\;<300{}^{\circ}:\;\left(R^{\prime },G^{\prime },B^{\prime }\right)=\left(X,0,C\right)$
If $300{}^{\circ}\;\leq H\;<360{}^{\circ}:\;\left(R^{\prime },G^{\prime },B^{\prime }\right)=(C,0,X)$

Finally, the output RGB values are obtained by adding *m* and scaling back to [0, 255] as represented in Equation (10):

(10) $$R=\left(R^{\prime }+m\right)\cdot 255,\;G=\left(G^{\prime }+m\right)\cdot 255,\;B=(B^{\prime }+m)\cdot 255$$

This complete transformation ensures that the image retains the visual enhancements from gamma correction while restoring the format required for further processing like thresholding and segmentation.

### 2.4. Segmentation

After preprocessing, the color-enhanced Pap smear images will be segmented. The image segmentation technique used in this algorithm is multi-Otsu thresholding. Otsu thresholding is a popular method in image processing that was introduced to determine an optimal threshold value that minimizes the intra-class variance (the variance within the same class) and maximizes the inter-class variance (the variance between different classes). The goal of Otsu thresholding is to determine the thresholds t_1_ and t_2_ that maximize the between-class variance $\sigma_{B}^{2}$ while minimizing the within-class variance $\sigma_{W}^{2}$. For a grayscale image with *L* intensity levels (0 to 255), and a probability distribution of pixel intensities given by the normalized histogram *p*(*i*), the total variance is:

(11) $$\sigma_{T}^{2}=\;\sum_{i=0}^{L-1}{\left(i-\mu_{T}\right)}^{2}p(i)$$

where $\mu_{T}=\sum_{i=0}^{L-1}i\cdot p(i)$ is the global mean.

**Figure 5:**
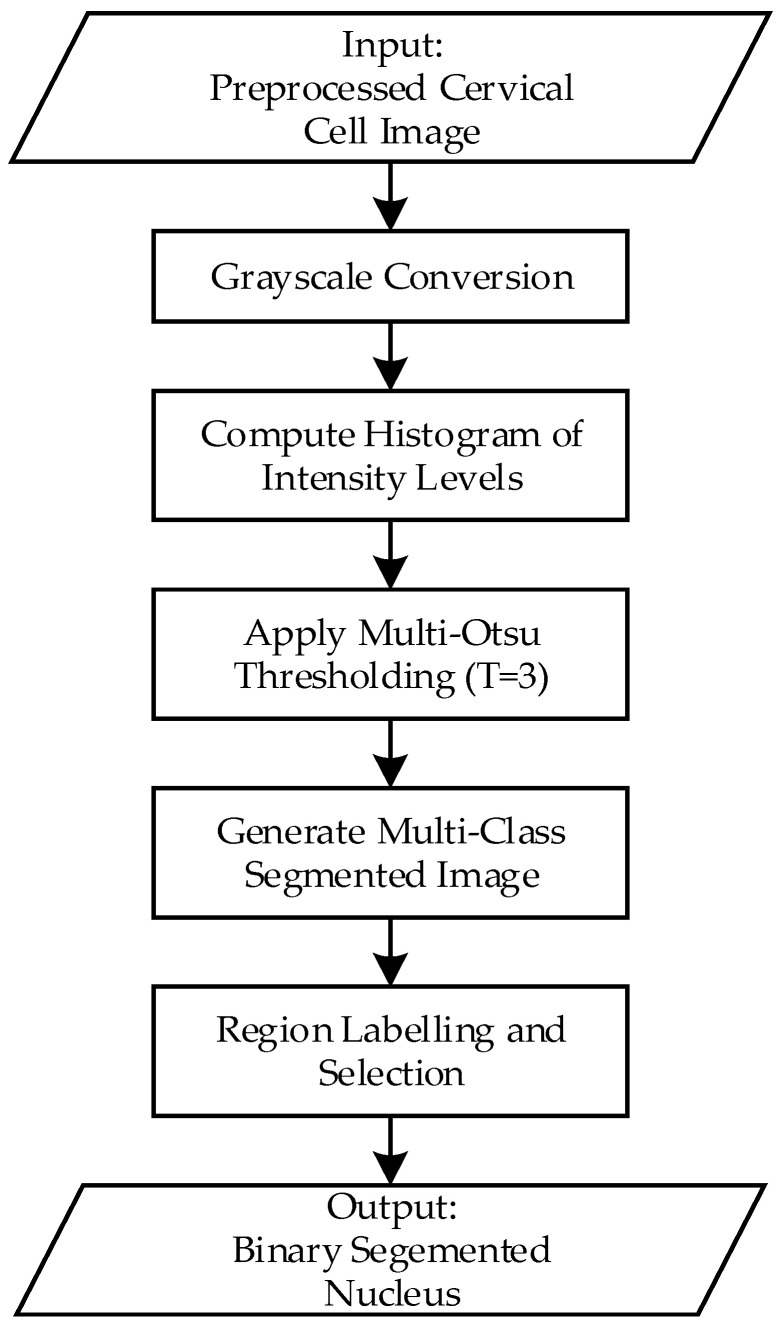
Flowchart of the multi-Otsu thresholding process for cervical nucleus segmentation.

However, traditional Otsu thresholding is limited to binary segmentation. In cases where more than two regions need to be segmented, such as separating nuclei, cytoplasm, and background in cervical Pap smear images, an extension of this method called multi-Otsu thresholding is used. Figure 5 illustrates the step-by-step implementation of the multi-Otsu thresholding process applied in this study.

First, the preprocessed images will be converted to grayscale. This simplifies the data by reducing it to a single channel, representing the intensity values. Next, the grayscale image’s intensity distribution is analyzed by computing its histogram. The histogram indicates the frequency of each intensity value, revealing the overall distribution of brightness in the image. In addition, the algorithm calculates the optimal threshold values that divide the intensity range into multiple classes. Accordingly, the algorithm evaluates all possible combinations of threshold values to minimize the variance within each class (intra-class variance) and maximize the variance between classes (inter-class variance). In this project, with a three-class segmentation scenario, the algorithm computes two thresholds, *t*_1_ and *t*_2_, that divide the pixel intensities into three distinct regions. The pixel intensities are assigned to different classes based on the calculated threshold, three classes are defined as:

- Class 1: [0, t_1_]
- Class 2: [t_1_ + 1, t_2_]
- Class 3: [t_2_ + 1, L−1]

Each class $k\in \left\{1,2,3\right\}$ has:

(12) $${Class\;probability:\;\omega }_{k}=\;\sum_{i\in C_{k}}p\left(i\right)\;$$

(13) $${Class\;Means:\;\mu }_{k}=\sum_{i\in C_{k}}i\cdot p\left(i\right)\;$$

The between class variance is then calculated as:

(14) $$\sigma_{B}^{2}=\;\sum_{k=0}^{3}\omega_{k}{\left(\mu_{k}-\mu_{T}\right)}^{2}$$

Multi-Otsu selects thresholds (t_1_,t_2_) that maximize $\sigma_{B}^{2}$, which ensures that the classes are as distinct as possible in terms of intensity.

Next, segmentation is performed by assigning each pixel a unique label or intensity corresponding to its class. For visualization, these classes can be represented by distinct colors or shades of grey, allowing for easy differentiation between regions like nuclei, cytoplasm, and background. To isolate the nuclei (or other regions of interest), pixels belonging to Class 1 are extracted as a binary mask. In the binary mask, the extracted region is represented by white (1), while all other regions are represented by black (0), and this is the final output of this segmentation process.

Although multi-Otsu thresholding effectively segmented the image into distinct regions, the resulting binary image still exhibited certain issues. These included small noisy regions, incomplete or fragmented nuclei due to gaps within objects, and jagged or irregular boundaries that impacted the clarity of the segmentation. Thus, a series of post-processing steps were implemented to address these challenges and enhance the quality of the segmented image. These steps aimed to refine the binary image, remove artifacts, and ensure the accurate representation of nuclei regions for further analysis.

### 2.5. Post-Processing

**Figure 6:**
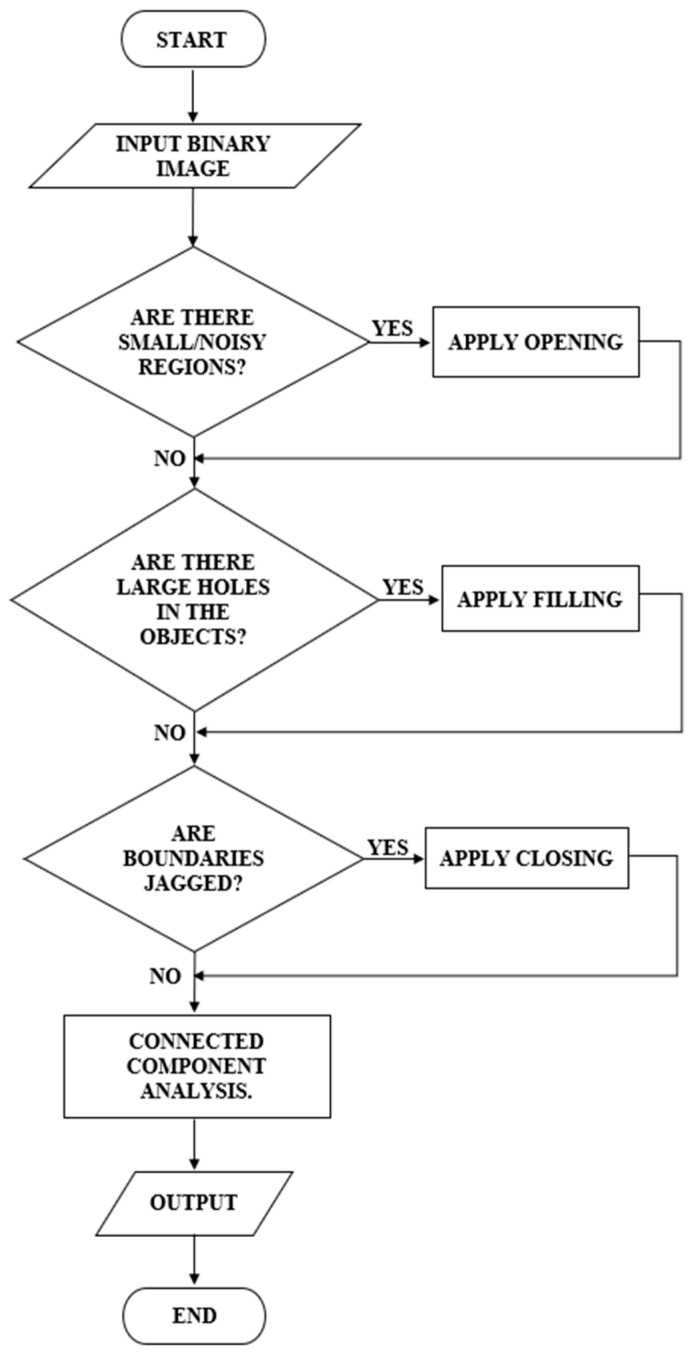
Post-processing Flowchart.

Figure 6 depicts a flowchart for post-processing in this algorithm. After the initial segmentation using multi-Otsu thresholding, the resulting binary image was subjected to connected component analysis to detect small noisy regions that were likely artifacts or irrelevant details.

Connected Component Analysis (CCA) labels and groups connected pixels in a binary image. Let $B\left(x,y\right)$ be a binary image such that:

(15) $$B\left(x,y\right)=\;\left\{\begin{array}{c} 1,\;if\;pixel\;belong\;to\;nucleus\\ 0,\;otherwise \end{array}\right.$$

Using 4-connectivity, the image is scanned and each group of connected pixels P_k_ is assigned a unique label:

(16) $$P=\;\overset{n}{\underset{k=1}{⋃}}P_{k},\;\;where\;P_{i}\cap P_{j}=\emptyset \;for\;i\neq j$$

Each component $P_{k}$ is then analyzed, and its area A_k_ is computed as:

(17) $$A_{K}=\;\sum_{(x,y)\in P_{k}}B(x,y)$$

Components with $A_{K}\;<\;A_{min}$ are discarded as noise.

If such regions were identified, morphological opening was applied, represented in Equation (18). This operation, involving erosion followed by dilation, effectively removed small objects while preserving the integrity of larger regions.

(18) I∘S=(I⊖S)⊕S

Subsequently, the binary image was evaluated for the presence of large holes within the segmented objects. If such gaps were detected, a filling operation was employed to close them, ensuring that the objects appeared solid and intact.

(19) $$I_{filled}=I\cup \left(H_{k}⊕S\right)\cap \bar{I}$$

The boundaries of the segmented regions were then examined for irregularities or jaggedness. If jagged boundaries were observed, morphological closing was used to smoothen the edges and bridge small gaps. Additionally, edge refinement was optionally applied to enhance boundary clarity. This involved detecting object edges using methods like Canny or Sobel, dilating these edges, and integrating them back into the binary image for better definition.

Finally, the post-processed binary image was filtered based on region size Where $A_{k}$ is the area of each region $P_{k}$, and regions below the threshold are removed as represented in Equation (20):

(20) $$I_{final}=\underset{A_{k}\;\geq \;A_{min}\;}{⋃}P_{k}$$

That is, regions smaller than a specified threshold were removed to eliminate any remaining noise, leaving only meaningful structures for further analysis. The resulting binary image was saved with enhanced clarity, filling gaps and smoother boundaries.

## 3. Results

### 3.1. Output Comparison

**Figure 7:**
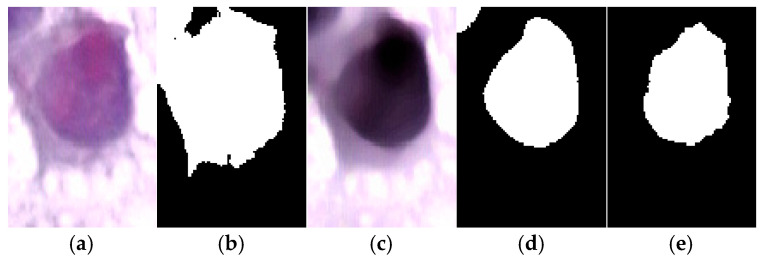
Comparison of segmentation results where (**a**) Original cervical cell image. (**b**) Direct Otsu thresholding on the original image, which fails to segment the nucleus properly compared to the ground truth. (**c**) Enhanced image produced by the proposed preprocessing pipeline. (**d**) Segmentation result using the proposed method, showing accurate delineation of the nucleus. (**e**) Ground truth segmentation mask.

To evaluate the necessity of the proposed preprocessing steps, we compared direct Otsu thresholding on the original image with segmentation using the enhanced image (Figure 7). As shown in Figure 7b, Otsu applied directly to the raw input fails to capture the nuclear boundaries accurately. In contrast, the enhanced image in Figure 7c combined with the proposed segmentation pipeline produces a result as shown in Figure 7d that closely matches the ground truth displayed in Figure 7e. This demonstrates that the preprocessing stages are essential to achieving accurate segmentation and confirms that the proposed framework is not an oversimplification, but a systematic enhancement over naïve thresholding.

To evaluate the effectiveness of the proposed segmentation algorithm, a comparative analysis was conducted against several established threshold-based segmentation methods, including Bradley, Feng, Niblack, Nick, and Sauvola, as summarized in Table 1. These methods were selected due to their widespread use in biomedical image segmentation, particularly for handling low-contrast and unevenly illuminated images, which are common characteristics of Pap smear slides. By comparing our method with these classical techniques, we aim to demonstrate its robustness and improved performance under challenging imaging conditions. Out of the 70 Pap smear images used for evaluation, three representative samples; selected based on varying degrees of cell overlap and staining quality are presented in Table 2 to visually illustrate the differences in segmentation outcomes.

### 3.2. Image Quality Assessment and Statistical Validation

To evaluate the performance of the proposed algorithms, a total of 70 output images were subjected to a comprehensive Image Quality Assessment (IQA). The evaluation employed both classification-based and image-quality-based metrics, namely Precision, Recall (Sensitivity), F1-score, Accuracy, and Peak Signal-to-Noise Ratio (PSNR). The mathematical definitions for these metrics are as shown in Equations (21)–(25):

- Precision:
  (21) $$Precision=\frac{TP}{TP+FP}.$$
  which measures the proportion of correctly identified nuclei pixels among all pixels predicted as nuclei. High Precision indicates fewer false positives.
- Recall (Sensitivity):
  (22) $$Recall=\frac{TP}{TP+FN}.$$
  indicates how many actual nuclei pixels were correctly detected. High Recall suggests minimal missed detections.
- F1-score:
  (23) $$F1=2\cdot \frac{Precision\cdot Recall}{Precision+Recall}.$$
  represents the harmonic mean of Precision and Recall, providing a balanced measure of performance.
- Accuracy:
  (24) $$Accuracy=\frac{TP+TN}{TP+TN+FP+FN}.$$
  reflects the overall correctness of classification across all pixels.

These metrics were computed across and the average results are presented in Table 3. The comparative performance highlights the effectiveness of the proposed method relative to commonly used segmentation techniques, including Bradley, Feng, Niblack, Nick, and Sauvola.

Precision, which indicates the proportion of correctly predicted nucleus pixels among all predicted positives, reached 99.65% for the proposed method—the highest among all techniques. In contrast, the second-highest value was observed in Sauvola (98.35%), while other methods like Niblack and Nick performed substantially lower (84.66% and 86.66%, respectively). This indicates that the proposed method rarely misclassifies background as nucleus, which is critical in minimizing false-positive detections in diagnostic applications.

The F-measure, a harmonic mean of Precision and Sensitivity, was 97.29% for the proposed method, outperforming all others. The next best was Bradley (95.97%), followed by Sauvola (93.86%). Lower F-measure scores in Feng (92.41%) and Niblack (90.14%) reflect less balanced segmentation outcomes. The high F-measure of the proposed method demonstrates its ability to maintain both high detection rates and low false-positive errors.

In terms of Sensitivity, the proposed method achieved 95.16%, indicating a high rate of true nucleus detection. While Nick and Niblack had slightly higher Sensitivity values (99.59% and 97.90%, respectively), their lower Precision scores show they over-classified nuclei, resulting in many false positives. Therefore, the proposed method strikes a more desirable balance between detecting true nuclei and avoiding misclassification.

Accuracy, which reflects the overall correctness of classification across all image pixels, was 98.39% for the proposed method—substantially higher than all competitors. Bradley achieved 94.02%, Sauvola 91.33%, and Feng only 89.11%. This result underscores the reliability of the proposed segmentation across the entire image, not just in isolated regions.

To evaluate the effectiveness of the proposed nucleus segmentation method, we compared it against several established adaptive thresholding methods, including Sauvola, Bradley, Nick, Niblack and Feng. While initial evaluations included all four baselines, Nick, Niblack and Feng methods consistently yielded performance metrics below the 90% threshold across key criteria, indicating relatively poor segmentation quality. Therefore, these two methods were excluded from further statistical testing.

Paired *t*-tests were conducted to compare the proposed method against the two most competitive methods, Sauvola and Bradley, both of which achieved metrics above 90% across all evaluation measures. The statistical analysis was based on 10 independent trials, assessing Precision, F1-score, Sensitivity, and Accuracy. Mean values with standard deviations, mean differences, and corresponding *p*-values are presented in Table 4 and Table 5. A significance level of *p* < 0.05 was applied to determine the statistical relevance of the observed improvements.

As shown in Table 4 and Table 5, the proposed segmentation method consistently outperformed both the Sauvola and Bradley methods across all evaluated metrics. When compared to the Sauvola method, the proposed method achieved higher Precision (98.98% vs. 97.58%), F1-score (96.61% vs. 93.50%), Sensitivity (95.61% vs. 91.09%), and Accuracy (96.58% vs. 93.57%), with statistically significant differences in each metric (*p* < 0.05). Similarly, against the Bradley method, the proposed method recorded substantially higher values for Precision (98.98% vs. 94.36%), F1-score (96.61% vs. 94.34%), Sensitivity (95.61% vs. 89.53%), and Accuracy (96.58% vs. 85.06%). The paired *t*-test confirmed that all these differences were statistically significant, with particularly strong significance observed for Precision and Accuracy (*p* = 0.0003). These results highlight the consistency and robustness of the proposed method across different evaluation criteria and trials.

To examine whether the 30:40 distribution of normal vs. abnormal images influenced performance, we computed segmentation metrics separately for the two groups as shown in Table 6. Normal images achieved slightly higher Accuracy (97.16% vs. 95.98%), while abnormal images achieved slightly higher Sensitivity (95.15% vs. 92.99%). Macro-averaged results (Precision 98.24%, F-measure 96.34%, Sensitivity 94.07%, Accuracy 96.57%) were nearly identical to the overall averages (99.65%, 97.29%, 95.16%, 98.39%). These findings indicate that the mild imbalance in our subset had no significant impact on algorithm performance.

To further contextualize the performance of the proposed enhanced thresholding method, we compared it against several state-of-the-art deep learning–based cervical cell segmentation approaches reported in the literature. Most of these studies employed the same Herlev dataset, albeit with different training epochs and hyperparameter settings according to each author’s empirical design (except Liu et al. which focused on tissue-level analysis). Since the primary objective of this work was to advance thresholding-based segmentation, we reproduced and evaluated classical thresholding methods in detail (Table 2 and Table 3), while adopting published results for deep learning methods to provide broader context, we did not reproduce or retrain those models ourselves, as their results are reported using the same Herlev dataset under each author’s own experimental settings. Nevertheless, to ensure fairness in direct comparison, we also implemented a baseline U-Net model using the same train–test split ratio and evaluation metrics as our method. Experiments were performed on both raw input images and pre-processed images, each trained for 30 epochs.

As presented in Table 7, the proposed enhanced thresholding method achieved the highest Precision (99.65%) and Accuracy (98.39%) among all evaluated approaches, while maintaining a strong Sensitivity of 95.16%. In contrast, the baseline U-Net model trained under our experimental settings yielded limited performance, with relatively low Precision (45.76%) despite moderate Sensitivity (89.77%), resulting in reduced overall Accuracy (78.41%). Incorporating the proposed preprocessing pipeline substantially improved U-Net performance, increasing Precision to 85.24% and Accuracy to 90.32%. Compared with published deep learning approaches on the Herlev dataset, the proposed method consistently demonstrated competitive or superior performance, particularly in Precision and Accuracy.

## 4. Discussion

This study presents a hybrid segmentation approach that achieves notable improvements in cervical cell nucleus segmentation, addressing long-standing challenges such as poor contrast, overlapping cells, and image artifacts. The superior performance observed across multiple quantitative metrics—particularly in Precision, F-measure, Accuracy, and PSNR—underscores the scientific significance of the method’s design.

The high segmentation quality can be attributed to the method’s adaptive framework, which dynamically responds to the heterogeneity of Pap smear images. Unlike conventional methods that rely on fixed parameters and global thresholding strategies, the proposed integration of adaptive gamma correction, multi-Otsu thresholding, and image-specific morphological refinement allows for more nuanced and localized image enhancement and segmentation. This ensures that nuclei are effectively isolated even in cases of low contrast or densely packed cell clusters.

From a scientific perspective, the balance achieved between high Sensitivity and very high Precision is particularly noteworthy. While some existing methods, such as Niblack and Nick, exhibit high Sensitivity, they suffer from increased false positives due to over-segmentation. In contrast, the proposed method maintains strong detection capability while significantly reducing background misclassification. This balance is critical in medical imaging, where overestimation or underestimation of cellular regions can adversely affect diagnostic accuracy.

The statistical significance of the improvements (*p* < 0.05) further supports the validity of the performance gains, ruling out the possibility that they occurred due to random variation. Although methods like Nick and Niblack were initially considered, their comparatively lower performance across multiple trials rendered them unsuitable for further statistical analysis. The superior results achieved by the proposed method can be attributed to its multi-layer clustering mechanism, which better adapts to the complex texture and illumination variations inherent in Pap smear images. Overall, these findings affirm the method’s potential as a reliable segmentation technique for cervical cell analysis in clinical and computer-aided diagnostic applications.

The observation that our method achieved higher performance compared to some deep learning approaches reported in the literature may seem unexpected, but this can be explained by several factors. First, the Herlev dataset is relatively small (fewer than 1000 images), which can limit the performance of deep learning models that generally benefit from large-scale annotated data. Our subset of images, selected for its challenging characteristics such as low quality, irregular nucleus shapes, and varying sizes, further emphasizes this limitation. Second, our approach incorporates preprocessing steps designed to enhance contrast and improve nucleus visibility, which likely contributed to better segmentation accuracy. Differences in preprocessing pipelines and input preparation can influence the final performance of any segmentation approach, whether traditional or deep learning-based. Third, deep learning models often require careful hyperparameter tuning, such as learning rate, optimizer settings, and network architecture choices, to achieve optimal results. In contrast, the proposed enhanced traditional method minimizes this complexity because the preprocessing phase standardizes image quality prior to segmentation, reducing variability and the need for iterative parameter adjustments. Finally, variations in evaluation methodologies across studies can also contribute to differences in reported metrics. While our evaluation focused on pixel-level accuracy and related measures, some studies may have emphasized region-based or edge-based criteria, making direct numerical comparison difficult.

Overall, these factors suggest that the observed differences are not solely due to the choice of method but also reflect variations in dataset size, preprocessing strategies, and evaluation protocols. Although the proposed approach achieves competitive performance compared with both traditional and deep learning–based methods, its evaluation was limited to the Herlev dataset. While Herlev is a widely used benchmark that ensures comparability and reproducibility, it reflects images acquired under standardized conditions. Methods that rely on pre-processing and unsupervised thresholding may be fragile when applied to images from different hospitals with varying staining or illumination.

Future work will address these limitations by validating the framework on multi-center datasets collected from diverse clinical environments. In addition, integration with lightweight machine learning strategies may further enhance segmentation robustness while maintaining computational efficiency and interpretability.

## 5. Conclusions

This study presented an adaptive nucleus segmentation method for Pap smear images by integrating gamma correction, multi-Otsu thresholding, and morphological post-processing. The proposed approach significantly outperformed conventional methods in terms of accuracy, precision, and overall segmentation quality. These findings demonstrate that carefully optimized traditional image processing techniques can still deliver competitive performance for medical image analysis, particularly in challenging imaging conditions. A limitation of this work is that the evaluation was conducted on 70 carefully selected images, curated in consultation with expert pathologists from Hospital Tuanku Fauziah, Kangar, Malaysia. Only diagnostically relevant and high-quality samples were retained, while low-quality or artifact-prone images were excluded to ensure reliability. Although this curated set provides a strong proof-of-concept validation, the relatively small number of images may restrict the generalizability of the results.

Future work will therefore focus on expanding the dataset to include larger and more diverse collections of Pap smear images, validating the approach across different imaging conditions, and exploring integration into automated classification pipelines or hybrid frameworks with machine learning and deep learning for comprehensive cervical cancer screening support.

## Figures and Tables

**Table 1:** Comparison of various image segmentation approaches commonly used in medical image processing, including their mathematical formulations and limitations.

Method	Key Principles	Mathematical Formulation	Limitation
Bradley	Adaptive thresholding compares each pixel intensity to the average of its surrounding neighborhood to handle uneven illumination. It uses an integral image for fast, constant-time computation of local averages across rectangular regions. The method involves two passes: first to build the integral image, and second to classify each pixel as dark or light based on whether its intensity falls below a set percentage of the local average.	$T=m\left(1-\frac{k}{100}\right)$ m = mean value and k = 12 [17].	Not capable of solving extreme illumination problems.
Feng	Local adaptive thresholding technique that enhances segmentation accuracy by computing both the local mean and standard deviation within a sliding window around each pixel. This dual-statistic approach allows the threshold to dynamically adjust based on local brightness and contrast.	$T=\left(1-\alpha 1\right)\times m+\alpha 2\times \left(\frac{s}{R_{s}}\right)\times \left(m-M\right)+\alpha 3\times M$ Rs = dynamic range of gray value standard deviation, m = mean value, s = standard deviation, α = coefficient, M = minimum value of the gray levels [17].	Sensitive to gradient noise, which may lead to errors in highly textured or low-contrast areas.
Niblack	Local adaptive thresholding algorithm that computes a pixel-wise threshold by analyzing the statistical properties of its surrounding neighborhood. It slides a window across the image to calculate the local mean and standard deviation, which are combined with a sensitivity constant to produce a dynamic threshold responsive to local brightness and contrast. This approach effectively handles non-uniform backgrounds and enhances the detection of dark features on lighter regions. The window size and sensitivity constant significantly affect its responsiveness to fine details and noise, making careful parameter tuning crucial for optimal results.	T=m+k·s m = mean value, s = standard, k = constant parameter that determines the weight of the standard deviation.	Produces noisy results in regions with high texture or uneven backgrounds.
Nick	The Nick method is a refined local adaptive thresholding algorithm that builds on Niblack by introducing a logarithmic adjustment to the standard deviation term, improving stability and reducing sensitivity to noise. It calculates the threshold for each pixel using local mean and standard deviation within a sliding window, but dampens the influence of high-variance regions to prevent over-segmentation. This contrast-aware adaptation allows smoother transitions between foreground and background, making it effective in images with uneven illumination and subtle features.	$\left(x,y\right)=m+k\sqrt{\frac{\left(I^{2}-m^{2}\right)}{N}}$ m = mean value, k = −0.13, I = pixel intensity and N = image size [17].	Can introduce errors in very small or highly contrasted regions, leading to suboptimal results.
Sauvola	Local adaptive thresholding algorithm that improves upon Niblack by introducing a normalization factor to the standard deviation, making the threshold more stable in noisy or low-contrast regions. It calculates the threshold for each pixel using the local mean and standard deviation within a sliding window, but scales the influence of the standard deviation relative to a predefined maximum value. This dynamic adjustment ensures that the threshold remains responsive to local contrast while preventing excessive sensitivity in homogeneous areas.	$T=m\left(1-k\left(1-\frac{s}{R}\right)\right)$ R = gray-level (128), m = mean value, s = standard deviation and k = 0.1 [17].	Struggles with extremely uneven backgrounds or excessively low contrast in specific image areas.

**Table 2:** Comparison of the segmentation results for three selected sample images using five different methods.

Class/Method	Bradley	Feng	Niblack	Nick	Sauvola	Proposed Method
Moderate Dysplasia	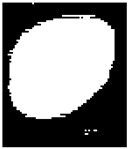	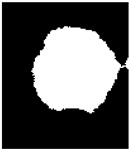	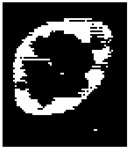	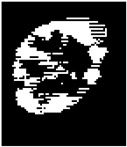	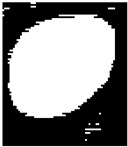	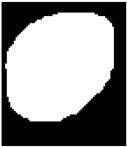
Severe Dysplasia	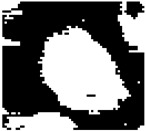	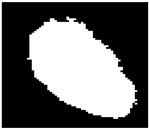	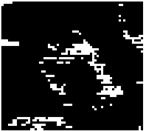	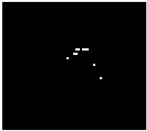	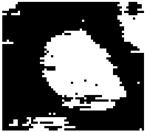	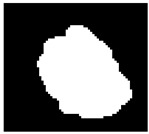
Superficial Squamous Epithelia	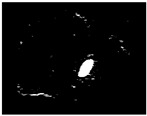	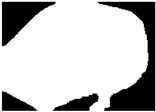	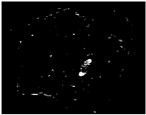	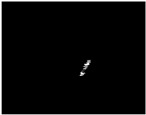	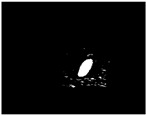	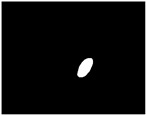

**Table 3:** Performance comparison of thresholding-based segmentation methods using evaluation metrics.

Method	Precision (%)	F-Measure (%)	Sensitivity (%)	Accuracy (%)
Bradley	95.00	95.96	97.41	94.02
Feng	98.28	92.40	88.10	89.11
Niblack	84.66	90.14	97.91	84.20
Nick	86.66	91.99	99.59	87.22
Sauvola	98.34	93.86	90.50	91.33
Proposed Method	99.65	97.29	95.16	98.39

**Table 4:** Comparison of segmentation performance between the proposed method and the Sauvola method (mean ± standard deviation, *n* = 10 trials). Statistical significance assessed by paired *t*-test (*p* < 0.05).

Metric	Proposed Method (Mean ± Std)	Sauvola Method (Mean ± Std)	Mean Difference	*p*-Value
Precision	98.98 ± 0.57	97.58 ± 1.63	1.4	0.0272
F1-Score	96.61 ± 0.83	93.50 ± 2.33	3.11	0.0023
Sensitivity	95.61 ± 0.67	91.09 ± 3.10	4.52	0.0024
Accuracy	96.58 ± 1.20	93.57 ± 3.13	3.01	0.0306

**Table 5:** Comparison of segmentation performance between the proposed method and the Bradley method (mean ± standard deviation, *n* = 10 trials). Statistical significance assessed by paired *t*-test (*p* < 0.05).

Metric	Proposed Method (Mean ± Std)	Bradley Method (Mean ± Std)	Mean Difference	*p*-Value
Precision	98.98 ± 0.57	94.36 ± 2.53	4.62	0.0003
F1-Score	96.61 ± 0.83	94.34 ± 2.53	2.27	0.039
Sensitivity	95.61 ± 0.67	89.53 ± 6.82	6.08	0.0191
Accuracy	96.58 ± 1.20	85.06 ± 6.39	11.53	0.0003

**Table 6:** Segmentation performance metrics for normal and abnormal cervical cell images (mean ± standard deviation). Groupwise (Normal *n* = 30, Abnormal *n* = 40) and macro-averaged results are reported to evaluate the effect of dataset class imbalance.

Class	Precision (%)	F-Measure (%)	Sensitivity (%)	Accuracy (%)
Normal (*n* = 30)	98.14 ± 1.08	96.77 ± 1.18	92.99 ± 1.69	97.16 ± 1.00
Abnormal (*n* = 40)	98.34 ± 1.20	95.91 ± 1.19	95.15 ± 1.17	95.98 ± 2.06
Macro Average	98.24	96.34	94.07	96.57
Overall Average	99.65	97.29	95.16	98.39

**Table 7:** Comparison of segmentation performance between the proposed method and representative deep learning–based cervical cell segmentation methods on the Herlev dataset.

Method	Precision (%)	Sensitivity (%)	Accuracy (%)
DeepCervix [30]	-	91.10	90.30
Jantzen et al. [31]	-	97.50	93.60
Liu et al. [32]	-	93.50	92.35
MSERLS [33]	94.23	91.82	-
DeepCNN1 [34]	94.61	95.59	-
HMLS [35]	93.81	92.34	-
U-Net	45.76	89.77	78.41
U-Net + Proposed Preprocessing	85.24	87.91	90.32
Proposed Method	99.65	95.16	98.39

## Data Availability

The dataset used in this study is available for download from the MDE-Lab website at the University of the Aegean: https://mde-lab.aegean.gr/index.php/downloads/ (accessed on 13 August 2025). The data are publicly accessible and were used in accordance with the terms provided by the original source.

## References

[1] Guimarães Y.M., Godoy L.R., Longatto-Filho A., Dos Reis R. (2022). Management of Early-Stage Cervical Cancer: A Literature Review. Cancers.

[2] Sausen D.G., Shechter O., Gallo E.S., Dahari H., Borenstein R. (2023). Herpes Simplex Virus, Human Papillomavirus, and Cervical Cancer: Overview, Relationship, and Treatment Implications. Cancers.

[3] Sung H., Ferlay J., Siegel R.L., Laversanne M., Soerjomataram I., Jemal A., Bray F. (2021). Global Cancer Statistics 2020: GLOBOCAN Estimates of Incidence and Mortality Worldwide for 36 Cancers in 185 Countries. CA Cancer J. Clin..

[4] Shi J., Wang R., Zheng Y., Jiang Z., Zhang H., Yu L. (2021). Cervical Cell Classification with Graph Convolutional Network. Comput. Methods Programs Biomed..

[5] Mustafa W.A., Halim A., Jamlos M.A., Idrus S.Z.S. (2020). A Review: Pap Smear Analysis Based on Image Processing Approach. J. Phys. Conf. Ser..

[6] Macios A., Nowakowski A. (2022). False Negative Results in Cervical Cancer Screening—Risks, Reasons and Implications for Clinical Practice and Public Health. Diagnostics.

[7] Schiffman M., de Sanjose S. (2019). False Positive Cervical HPV Screening Test Results. Papillomavirus Res..

[8] Selby K., Sedki M., Levine E., Kamineni A., Green B.B., Vachani A., Haas J.S., Ritzwoller D.P., Croswell J.M., Ohikere K. (2023). Test Performance Metrics for Breast, Cervical, Colon, and Lung Cancer Screening: A Systematic Review. J. Natl. Cancer Inst..

[9] Rasheed A., Shirazi S.H., Umar A.I., Shahzad M., Yousaf W., Khan Z. (2023). Cervical Cell’s Nucleus Segmentation through an Improved UNet Architecture. PLoS ONE.

[10] Wang P., Wang L., Li Y., Song Q., Lv S., Hu X. (2019). Automatic Cell Nuclei Segmentation and Classification of Cervical Pap Smear Images. Biomed. Signal Process. Control.

[11] Fujita H. (2020). AI-Based Computer-Aided Diagnosis (AI-CAD): The Latest Review to Read First. Radiol. Phys. Technol..

[12] Nahrawi N., Mustafa W.A., Kanafiah S.N.A.M., Ahmad W.K.W., Rohani M.N.K.H., Rahim H.A. (2021). A Novel Nucleus Detection on Pap Smear Image Using Mathematical Morphology Approach. J. Biomim. Biomater. Biomed. Eng..

[13] Plissiti M.E., Nikou C., Charchanti A. (2011). Automated Detection of Cell Nuclei in Pap Smear Images Using Morphological Reconstruction and Clustering. IEEE Trans. Inf. Technol. Biomed..

[14] Bataineh B. (2023). Brightness and Contrast Enhancement Method for Color Images via Pairing Adaptive Gamma Correction and Histogram Equalization. Int. J. Adv. Comput. Sci. Appl..

[15] Tan D.S., Mahmood D., Nisar H., Yeap K.H., Dakulagi V., Elaraby A. (2021). Comparison of Segmentation Performance of Activated Sludge Flocs Using Bright-Field and Phase-Contrast Microscopy at Different Magnifications. IOP Conf. Ser. Earth Environ. Sci..

[16] Mustafa W.A., Abdul-Nasir A.S., Mohamed Z. (2018). Malaria Parasites Segmentation Based on Sauvola Algorithm Modification. Malays. Appl. Biol..

[17] Ding Y., Yue W., Li Q. (2023). Automated Segmentation of Cervical Cell Images Using IMBMDCR-Net. Int. J. Mach. Learn..

[18] Mahyari T.L., Dansereau R.M. (2022). Multi-Layer Random Walker Image Segmentation for Overlapped Cervical Cells Using Probabilistic Deep Learning Methods. IET Image Process..

[19] Liu J., Fan H., Wang Q., Li W., Tang Y., Wang D., Zhou M., Chen L. (2022). Local Label Point Correction for Edge Detection of Overlapping Cervical Cells. Front. Neuroinform..

[20] Li X., Du M., Zuo S., Zhou M., Peng Q., Chen Z., Zhou J., He Q. (2023). Deep Convolutional Neural Networks Using an Active Learning Strategy for Cervical Cancer Screening and Diagnosis. Front. Bioinform..

[21] Ramakrishnan V., Artinger A., Barragan L.A.D., Daza J., Winter L., Niedermair T., Itzel T., Arbelaez P., Teufel A., Cotarelo C.L. (2024). Nuclei Detection and Segmentation of Histopathological Images Using a Feature Pyramidal Network Variant of a Mask R-CNN. Bioengineering.

[22] Zhao K., Niyogisubizo J., Xiao L., Pan Y., Wei D., Rosiyadi D., Wei Y. A Novel Deep Learning Approach Featuring Graph-Based Algorithm for Cell Segmentation and Tracking. Proceedings of the 2023 IEEE International Conference on Bioinformatics and Biomedicine (BIBM).

[23] Ren J., Che J., Gong P., Wang X., Li X., Li A., Xiao C. (2024). Cross Comparison Representation Learning for Semi-Supervised Segmentation of Cellular Nuclei in Immunofluorescence Staining. Comput. Biol. Med..

[24] Khiruddin K.D.N., Mustafa W.A., Jamaludin K.R., Ab Rahman K.S., Alquran H., Junaini S. (2025). Automated Cervical Cell Nuclei Segmentation Based on Multilayer Unsupervised Clustering Algorithm and Morphological Approach. Mitt. Klost..

[25] Alias N.A., Mustafa W.A., Jamlos M.A., Ismail S., Alquran H., Rohani M.N.K.H. (2023). Pap Smear Image Analysis Based on Nucleus Segmentation and Deep Learning—A Recent Review. J. Adv. Res. Appl. Sci. Eng. Technol..

[26] Alias N.A., Mustafa W.A., Jamlos M.A., Nasrudin M.W., Mansor M.A.S., Alquran H. (2022). Edge Enhancement and Detection Approach on Cervical Cytology Images. J. Adv. Res. Appl. Sci. Eng. Technol..

[27] Fang M., Fu M., Liao B., Lei X., Wu F.X. (2024). Deep Integrated Fusion of Local and Global Features for Cervical Cell Classification. Comput. Biol. Med..

[28] Anandavally P.S.N., Bai V.M.A. (2024). Deep Neural Network for the Detection and Classification of Spontaneous Abortion Associated with Cervical Cancer. J. Adv. Res. Appl. Sci. Eng. Technol..

[29] Wubineh B.Z., Rusiecki A., Halawa K. (2024). Classification of Cervical Cells from the Pap Smear Image Using the RES_DCGAN Data Augmentation and ResNet50V2 with Self-Attention Architecture. Neural Comput. Appl..

[30] Chowdary G.J., Suganya G., Premalatha M., Yogarajah P. (2023). Nucleus Segmentation and Classification Using Residual SE-UNet and Feature Concatenation Approach in Cervical Cytopathology Cell Images. Technol. Cancer Res. Treat..

[31] Jantzen J., Dounias G. The Pap Smear Benchmark: Intelligent and Nature-Inspired Approaches in Pap Smear Diagnosis. Proceedings of the Special Session Proceedings of the NISIS 2006 Symposium, Puerto de la Cruz.

[32] Liu W., Li C., Xu N., Jiang T., Rahaman M.M., Sun H., Wu X., Hu W., Chen H., Sun C. (2022). CVM-Cervix: A Hybrid Cervical Pap-Smear Image Classification Framework Using CNN, Visual Transformer and Multilayer Perceptron. Pattern Recognit..

[33] Lu Z., Carneiro G., Bradley A.P. (2015). An Improved Joint Optimization of Multiple Level Set Functions for the Segmentation of Overlapping Cervical Cells. IEEE Trans. Image Process..

[34] Hoi S.C.H., Jin R., Zhu J., Lyu M.R. Batch Mode Active Learning and Its Application to Medical Image Classification. Proceedings of the 23rd International Conference on Machine Learning.

[35] Braga A.M., Marques R.C.P., Medeiros F.N.S., Neto J.F.S.R., Bianchi A.G.C., Carneiro C.M., Ushizima D.M. (2021). Hierarchical Median Narrow Band for Level Set Segmentation of Cervical Cell Nuclei. Measurement.

